# Preparation of Al_2_O_3_/Ti(C,N)/ZrO_2_/CaF_2_@Al(OH)_3_ Ceramic Tools and Cutting Performance in Turning

**DOI:** 10.3390/ma12233820

**Published:** 2019-11-21

**Authors:** Zhaoqiang Chen, Shuai Zhang, Runxin Guo, Lianggang Ji, Niansheng Guo, Qi Li, Chonghai Xu

**Affiliations:** 1 School of Mechanical and Automotive Engineering, Qilu University of Technology (Shandong Academy of Sciences), Jinan 250353, China; 2 Key Laboratory of Advanced Manufacturing and Measurement and Control Technology for Light Industry in Universities of Shandong, Qilu University of Technology (Shandong Academy of Sciences), Jinan 250353, China

**Keywords:** ceramic tool, ZrO_2_ whisker, coating, cutting performance, wear mechanisms

## Abstract

Aiming at the contradiction between the lubricating performance and mechanical performance of self-lubricating ceramic tools. CaF_2_@Al(OH)_3_ particles were prepared by the heterogeneous nucleation method. An Al_2_O_3_/Ti(C,N) ceramic tool with CaF_2_@Al_2_(OH)_3_ particles and ZrO_2_ whiskers was prepared by hot press sintering (frittage). The cutting performances and wear mechanisms of this ceramic tool were investigated. Compared with the Al_2_O_3_/Ti(C,N) ceramic tool, the Al_2_O_3_/Ti(C,N)/ZrO_2_/CaF_2_@Al(OH)_3_ ceramic tool had lower cutting temperatures and surface roughness. When the cutting speed was increased from 100 m/min to 300 m/min, a lot of CaF_2_ was smeared onto the surface of the ceramic tool, and the flank wear of the Al_2_O_3_/Ti(C,N)/ZrO_2_/CaF_2_@Al(OH)_3_ ceramic tool was reduced. The main wear mechanisms of the Al_2_O_3_/Ti(C,N)/ZrO_2_/CaF_2_@Al(OH)_3_ ceramic tool were adhesive wear and micro-chipping. The formation of solid lubricating film and the improvement of fracture toughness by adding ZrO_2_ whiskers and CaF_2_@Al(OH)_3_ were important factors for the Al_2_O_3_/Ti(C,N)/ZrO_2_/CaF_2_@Al(OH)_3_ ceramic tool to have better cutting performances.

## 1. Introduction

As a green manufacturing technology, dry cutting not only avoids the environmental pollution caused by cutting fluid, but also greatly reduces production costs [1]. However, due to the lack of lubricating fluid, the cutting temperature under dry cutting will increase, which will reduce the cutting performance and service life of the tool [2]. Ceramic tools have high hardness, high heat resistance, good chemical stability and good adhesion resistance; it is the main cutting tool for dry cutting [3,4]. Solid lubricants (such as CaF_2_, MoS_2_, h-BN, WS_2_, Mo, etc.) are added to the ceramic matrix, which can realize the self-lubrication of ceramic tools and reduce the wear of ceramic tools [5,6,7,8,9]. However, the mechanical properties of ceramic tools are reduced with the addition of lubricants, which limits the ultimate cutting performance of ceramic tools [10,11]. Therefore, how to make the tool material balance the lubrication performance and mechanical properties in dry cutting is the problem nowadays.

In recent years, with the development of surface coating technology, many scholars have found that coating on the surface of particles can impart new physical and chemical properties to the coated particles [12,13]. Chen et al. [14] prepared an Al_2_O_3_/TiC ceramic tool with a core-shell structure (h-BN)/SiO_2_, and the results show that the fracture toughness and bending strength of the tool material are improved. 

Zhang et al. [15] prepared Al_2_O_3_-coated h-BN powder by heterogeneous nucleation, compared with a Si_3_N_4_/TiC/h-BN ceramic tool directly added with h-BN, the Si_3_N_4_/TiC/ h-BN@Al_2_O_3_ ceramic tool has better anti-friction performance and wear resistance. In Wu et al.’s [16] research, they coated a layer of Ni on the surface of CaF_2_. Research shows that a Ni metal shell can effectively avoid the reaction of CaF_2_ and Al_2_O_3_ at high temperature. Compared with an Al_2_O_3_/(W,Ti)C ceramic tool, an Al_2_O_3_/(W,Ti)C/CaF_2_@Ni ceramic tool had better cutting performance and a lower friction coefficient. Therefore, adding coated solid lubricant particles to the ceramic tool material matrix can compensate for the loss of the mechanical properties of the ceramic tool and improve the wear resistance of the ceramic tool in dry cutting.

The application of whiskers on ceramic materials provides a new way for the toughening and strengthening of ceramic materials. Deng et al. [17] prepared an Al_2_O_3_/TiB_2_/SiC_w_ ceramic tool. Cutting tests have shown that the addition of SiC whiskers improves the wear resistance of ceramic tools. Bai et al. [18] introduced SiC whiskers into the ZrB_2_ base layer of the laminated ZrB_2_/BN ceramics, and found that the bridging and extraction of whiskers plays an important role in improving the fracture toughness. For the phase change toughened ceramic materials, the most typical example is ZrO_2_ whiskers [19]. Tuan et al. [20] prepared Al_2_O_3_/(t-ZrO_2_ + m-ZrO_2_) composite material, which is twice as tough as Al_2_O_3_ alone. To sum up, whiskers play an important role in improving the toughness of ceramic materials. Adding whiskers and coated solid lubricant particles to ceramic tool materials at the same time has an important application prospect for improving the mechanical properties and cutting performance of the ceramic tool. 

In order to solve the problem that the mechanical properties of ceramic tool materials were obviously reduced, we prepared the CaF_2_@Al(OH)_3_ particles by heterogeneous nucleation method. It was added to the Al_2_O_3_/Ti(C,N) ceramic matrix together with a ZrO_2_ whisker. Surface coating technology and whisker toughening are combined to improve the mechanical and cutting properties of the ceramic tool. An Al_2_O_3_/Ti(C,N) ceramic tool with CaF_2_@ Al_2_(OH)_3_ particles and ZrO_2_ whiskers was prepared by hot pressing sintering. Dry cutting tests were carried out on 40Cr hardened steel. The cutting performance and wear mechanism were studied and discussed through comparison with the Al_2_O_3_/Ti(C,N) ceramic tool.

## 2. Materials and Methods 

### 2.1. Preparation of Ceramic Tool Materials

**Figure 1:**
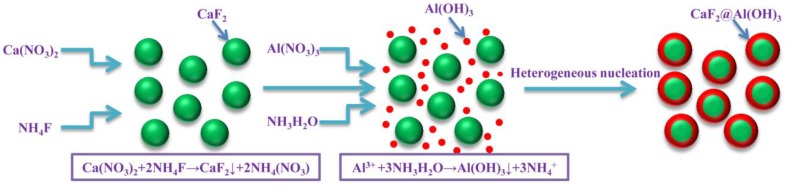
Schematic diagram of CaF_2_@Al(OH)_3_ preparation.

The preparation process of CaF_2_@Al(OH)_3_ particles is shown in Figure 1. NH_4_F and Ca(NO_3_)_2_ were dissolved in a mixed solvent that with a volume ratio of ethanol, benzene and water of 6:2:1 respectively, and ultrasonic stirring was carried out for 20 min to prepare NH_4_F solution (concentration of 0.22 mol/L) and Ca(NO_3_)_2_ solution (concentration of 0.1 mol/L), respectively. Then, under the condition of ultrasonic stirring, NH_4_F solution was slowly poured into Ca(NO_3_)_2_ solution to react for 5 minutes. Subsequently, the reaction product was centrifuged, washed and dried to obtain CaF_2_ particles. Al(NO_3_)_3_ was dissolved in a mixed solvent with a volume ratio of ethanol, benzene and water of 6:2:1 (the concentration of Al^3+^ was 0.3 mol/L), then PVP (the concentration was 5 g/L) and CaF_2_ (the concentration was 0.1 mol/L) prepared above were added, and ultrasonic stirring was maintained all the time. Then dilute ammonia water (the volume ratio of ethanol to ammonia water was 3:1) was added drop by drop to adjust the pH value to 7.0; the reaction temperature was controlled to be 25 °C, so that the reaction product Al(OH)_3_ formed a coating layer on the surface of CaF_2_, and finally the prepared CaF_2_@Al(OH)_3_ was centrifuged, cleaned and dried to obtain CaF_2_@Al(OH)_3_ particles.

Al_2_O_3_/Ti(C,N) were used as the matrix materials, and the average particle diameters were 200 nm and 80 nm, respectively. CaF_2_@Al(OH)_3_ and ZrO_2_ whiskers were used as the additive phases. CaF_2_@Al(OH)_3_ particle size was at an average of about 20–30 nm. The average diameter of ZrO_2_ whiskers were 1–3 μm. To ensure the phase change characteristics of ZrO_2_ whiskers, ZrO_2_ whiskers were doped with 3% Y_2_O_3_. MgO were used as a sintering assistant. 

Since Al(OH)_3_ decomposes into Al_2_O_3_ during sintering, the final result of hot pressing sintering was an Al_2_O_3_/Ti(C,N) ceramic tool with CaF_2_@Al_2_O_3_ and ZrO_2_. The ZrO_2_ volume content was 6% and the CaF_2_@Al_2_O_3_ volume content was 10%. In addition, an Al_2_O_3_/Ti(C,N) ceramic tool without ZrO_2_ and CaF_2_@Al(OH)_3_ was prepared under the same experimental conditions.

### 2.2. Performance Test of Ceramic Tool Materials 

After rough grinding, fine grinding and polishing, the ceramic sample material was processed into strips with a cross section of 3 mm × 4 mm × 35 mm. A Vickers hardness tester (Songlang electronic instrument co., Chongqing, China) was used to measure the hardness. The indentation load was 196 N and the pressure was kept at 15 s. Indentation was also used to tested the fracture toughness of sample materials. The flexural strength of the ceramic materials was tested by the three-point bending method, with a span of 20 mm and a loading rate of 0.5 mm/min. X-ray diffraction (XRD) (Bruker AXS Co., Karlsruhe, Germany) was used to detect the phase composition of the ceramic tool materials. Energy-dispersive X-ray spectroscopy (EDS) was used to analyze the phase of the ceramic tool. Scanning electron microscope (SEM) (Carl Zeiss Group, Oberkochen, Germany) was used to observe the surface of powder and the microstructure of the ceramic tool. 

### 2.3. Cutting Test of Ceramic Tool Materials

**Figure 2:**
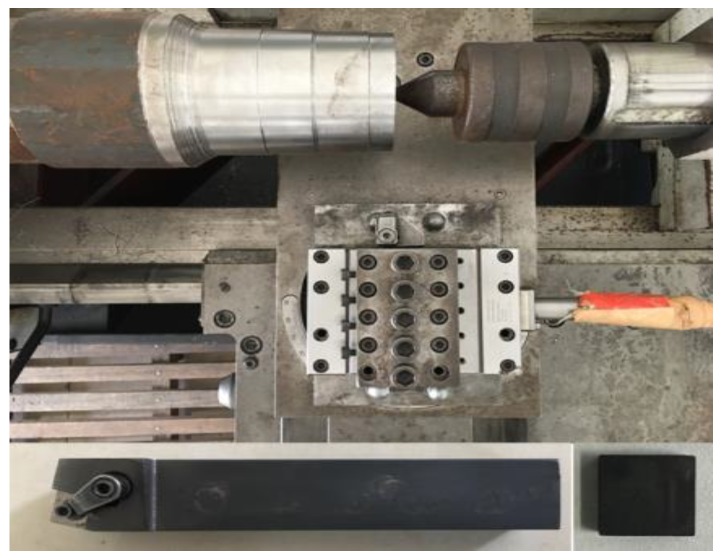
Photographs of ceramic tool, tool holders and test benches.

In this study, 40Cr steel (Hardness: 48-50 HRC) was used as the workpiece material, and its chemical composition is listed in Table 1. The ceramic tool, tool holders and test benches are shown in Figure 2. The ceramic tool geometry parameter mainly includes: clearance angle α_0_ = 5°, inclination angle λ_S_ = 0°, rake angle γ_0_ = 5°, side cutting edge angle k_r_ = 45° and the chamfering width b_r1_ = 0.1 mm. The machine model used was CDE6140A in the cutting test, and the model of the tool holder was Kenner GSSN R/L 2525M12-MN7 (Kennametal Inc., Latrobe, PA, USA). The cutting temperature in the cutting process was measured by an infrared thermal imager (model Flir-A320, FLIR Systems Inc., Portland, OR, USA). After the cutting distance of the tool reaches 500 m, the maximum value of the tip temperature was selected to compare the cutting temperatures. Measuring method of flank wear: a microscope was used to observe the wear of flank after the cutting test, and the wear of flank was read according to the scale. The tool failure standard was VB = 0.3 mm. Under given conditions, each test was replicated three times to eliminate the human error. The TR200 surface roughness measuring instrument (Time Group Inc., Jinan, China) was used to measure the surface roughness of the workpiece. Three different points were taken for each measurement and the average value was taken as the result. The surface roughness measure used in the paper was the arithmetic mean value of the surface roughness of profile, Ra.

## 3. Results and Discussion

### 3.1. Mechanical Properties and Microstructure of Ceramic Tool Materials

For the convenience of illustration, the Al_2_O_3_/Ti(C,N) ceramic tool material was recorded as ATCN, and the Al_2_O_3_/Ti(C,N) ceramic tool material with 10% CaF_2_@Al(OH)_3_ solid lubricant and 6% ZrO_2_ whisker was recorded as ATCN-Z-C in the following paper.

Table 2 lists the mechanical properties of the ATCN and ATCN-Z-C ceramic tools. In general, the addition of CaF_2_ will reduce the mechanical properties of this ceramic tool [10]. However, there are different conclusions for the ATCN-Z-C ceramic tool. Compared with ATCN ceramic tools, the ATCN-Z-C ceramic cutting tools had obviously decreased in hardness, but the bending strength was basically the same as that of the ATCN ceramic tools, and the fracture toughness was increased by 19.27% compared with those ATCN ceramic tools. This is mainly due to the existence of an Al_2_O_3_ shell which can improve the bonding strength between lubricant and matrix, and the addition of CaF_2_@Al(OH)_3_ can form an in-crystal structure in ceramic crystals [21,22].

**Figure 3:**
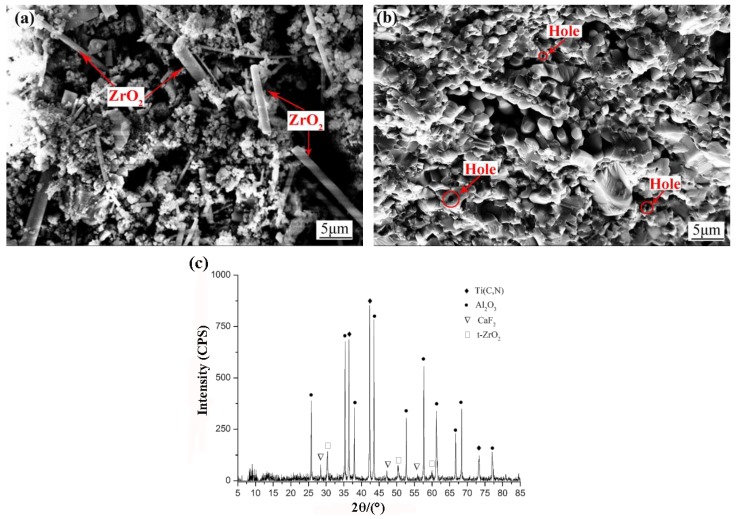
(**a**) Pre-sintered powder; (**b**) fracture surface and (**c**) XRD detection diagram of the ATCN-Z-C ceramic tool material.

Figure 3a shows pre-sintered powder of ATCN-Z-C ceramic tools. It is found that ZrO_2_ whiskers had uniform size and good dispersion effect in matrix materials. At the same time, it can be seen that other materials (Al_2_O_3_, Ti(C,N), CaF_2_@Al(OH)_3_) were basically in the nano scale. Figure 3b shows the fracture surface of ATCN-Z-C ceramic tools. It can be found that the density of the material was good, the grain of the matrix material was not abnormally grown. The hole from which the whiskers were pulled out can be observed from the Figure 3b. The extraction of whiskers will consume more energy, which is conducive to improving the fracture toughness of the ATCN-Z-C ceramic tool materials. The steps of transgranular fracture can also be found in Figure 3b. Therefore, the fracture mode of ATCN-Z-C ceramic tool was transgranular fracture and intergranular fracture, which was also beneficial to improve the fracture toughness of the ATCN-Z-C ceramic tool material. As shown in the Figure 3c is the XRD detection diagram of ATCN-Z-C ceramic tool. It can be seen from the figure that the characteristic peaks of Al_2_O_3_ and Ti(C,N) were obvious, and the characteristic peaks of CaF_2_ and ZrO_2_ can also be observed. It shows that the components of the ceramic cutting tool material had no chemical reaction in the hot pressing sintering process and have better chemical compatibility.

### 3.2. Cutting Performance

**Figure 4:**
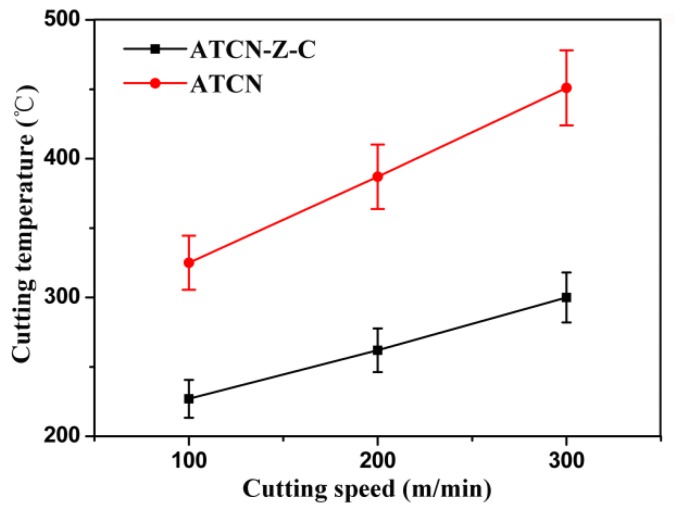
Cutting temperature of ATCN and ATCN-Z-C ceramic tools at cutting speed of 100, 200 and 300 m/min. (Test conditions: depth of cut α*_p_* = 0.2 mm, feed rates *f* = 0.102 mm/r).

The cutting temperatures were tested after the stable cutting 40Cr reached 500 m. The test results are shown in Figure 4. It can be found that the cutting temperature increases with the increase of the cutting speed. The ATCN-Z-C ceramic tool had lower cutting temperatures than the ATCN ceramic tool. When the cutting speeds were 100, 200 and 300 m/min, respectively, the cutting temperature of the ATCN-Z-C ceramic tool were 29.89%, 31.55% and 32.53% lower than the ATCN ceramic tool. At the same time, it can be found that the slope of the curve of the ATCN-Z-C ceramic tool was smaller than that of the ATCN ceramic tool. This shows that with the increase of cutting speed, the cutting temperature of the ATCN ceramic tool increases rapidly, while the ATCN-Z-C ceramic tool was relatively flat. It can be predicted, that as the cutting speed continues to rise, the difference between the cutting temperatures of the ATCN ceramic tool and the ATCN-Z-C ceramic tool will become larger and larger. The addition of ZrO_2_ whiskers and CaF_2_@Al(OH)_3_ reduced the cutting temperature of the ceramic tool. The higher cutting temperature of the ATCN ceramic tool will lead to faster wear of this same ATCN ceramic tool. The lower cutting temperature of the ATCN-Z-C ceramic tool was mainly due to the formation of lubricating film with CaF_2_ as the main body, and the improvement of the fracture toughness of the ceramic tool.

**Figure 5:**
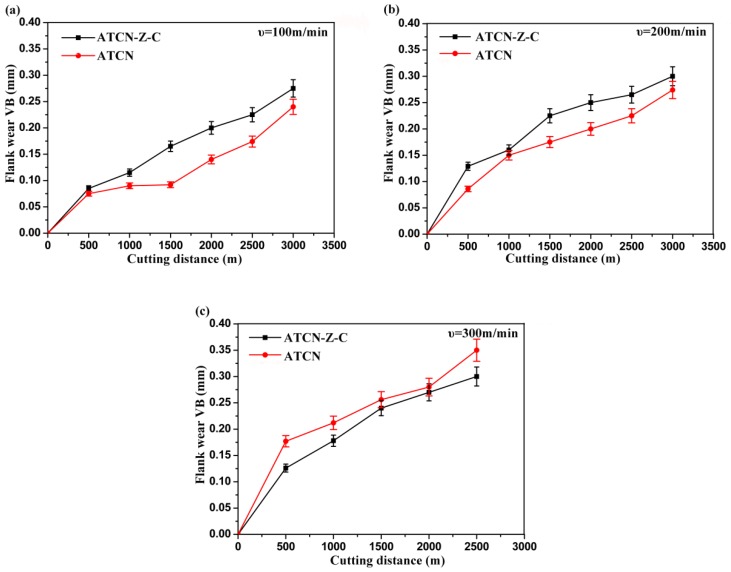
Flank wear of ATCN and ATCN-Z-C ceramic tools at cutting speed of (**a**) 100; (**b**) 200 and (**c**) 300 m/min. (Test conditions: depth of cut α*_p_* = 0.2 mm, feed rates *f* = 0.102 mm/r).

The flank wear of the ATCN ceramic tool and the ATCN-Z-C ceramic tool at different cutting speeds are shown in Figure 5. The results show that the flank wear of the ATCN ceramic tool and the ATCN-Z-C ceramic tool increases with the increase of cutting speed. At cutting speeds of 100 and 200 m/min, the ATCN ceramic tool had better wear resistance than the ATCN-Z-C ceramic tool because CaF_2_ does not easily form lubricating films. When the cutting speed reaches 300 m/min, the ATCN-Z-C ceramic tool exhibits better performance than the low speed cutting. The cutting ability and the flank wear were slightly less than the ATCN ceramic tool, which had better anti-friction and wear resistance. A similar conclusion was reached in Deng et al.’s research [23]. They found that with the increase of cutting speed, the friction coefficient of ceramic materials added with CaF_2_ was smaller. This was mainly because, with the increase of cutting speed, the cutting temperature also increases; thus CaF_2_ changes from brittle state to plastic state, and it was easier to drag on the surface of the ceramic tool to form a solid lubricating film. Therefore, the ATCN-Z-C ceramic tool was more suitable for cutting at higher cutting speeds.

**Figure 6:**
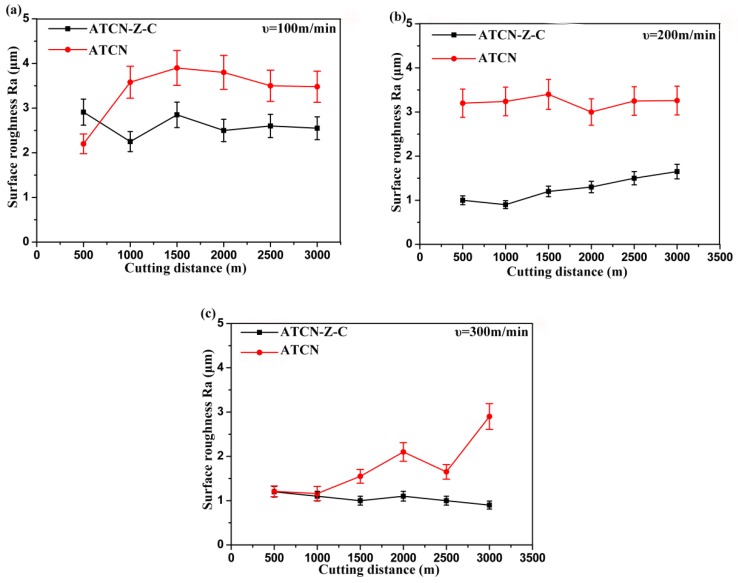
Surface roughness of ATCN and ATCN-Z-C ceramic tools at cutting speed of (**a**) 100; (**b**) 200 and (**c**) 300 m/min. (Test conditions: depth of cut α*_p_* = 0.2 mm, feed rates *f* = 0.102 mm/r).

Surface roughness is one of the methods to evaluate product precision and plays an important role in predicting processing performance. As shown in Figure 6, generally speaking, the value Ra of the surface roughness decreases with the increase of the cutting speed. This shows that with the increase of cutting speed, the surface qualities of the two ceramic tools were improved. For the ATCN ceramic tool, the value Ra of surface roughness reached 4 μm at a cutting speed of 100 m/min. When the cutting speed was increased to 300 m/min and the cutting distance was 1500 m, the wear of the ATCN ceramic tool will accelerate, resulting in a sudden increase in the Ra value of surface roughness. Subsequently, due to the oxidation reaction of Ti(C,N) at a higher cutting temperature, partial wear was repaired, which resulted in a reduction of the surface roughness value Ra at a cutting distance of 2000 m to 2500 m. Generally speaking, the surface roughness of the ATCN ceramic tool fluctuates greatly. In contrast, the surface roughness Ra of the ATCN-Z-C ceramic tool at different cutting speeds was less than 3 μm. Especially in high-speed cutting (300 m/min), the value Ra of the surface roughness was kept between 0.7 and 1.5 μm with little fluctuation. The analysis shows that the existence of solid lubricating film and the toughening effect of the ZrO_2_ whisker can reduce tool wear and improve the quality of the machined surface.

### 3.3. Wear Profile of Ceramic Tools and Its Antifriction Mechanism

**Figure 7:**
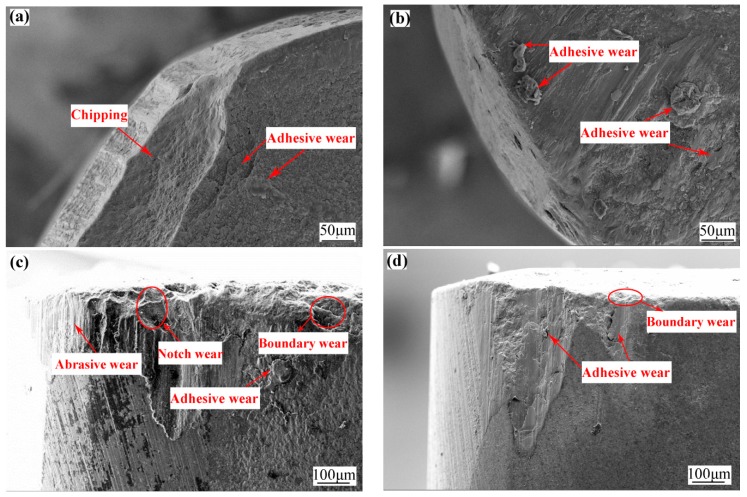
Wear profile of the rake faces of the (**a**) ATCN and (**b**) ATCN-Z-C ceramic tools, the flank face of (**c**) ATCN and (**d**) ATCN-Z-C ceramic tools. (Test conditions: depth of cut α*_p_* = 0.2 mm, feed rates *f* = 0.102 mm/r, cutting speed υ = 300 m/min)

Figure 7a,b show the wear profile of the rake faces of the ATCN ceramic tool and the ATCN-Z-C ceramic tool. It can be found that the tool tip and cutting edges of the ATCN ceramic tool were broken down, while the ATCN-Z-C was relatively light, which might be attributed to its relatively lower fracture toughness than that of the ATCN-Z-C ceramic tool (see Table 1). In addition, adhesion wear can be observed on the rake face of the ATCN ceramic tool and the ATCN-Z-C ceramic tool. 

Analysis shows that during the cutting process, serious friction occurs in the contact area between the tool and chip, and the cutting heat and cutting force lead to adhesive wear on the rake face of the ATCN ceramic tool and the ATCN-Z-C ceramic tool. At the same time, the existence of solid lubricant film can also be observed on the rake face of the ATCN-Z-C ceramic tool.

The flank wear of the ATCN ceramic tool is shown in Figure 7c. The flank wear of the ATCN ceramic tool was mainly notch wear and boundary wear. Abrasive wear and adhesive wear can also be observed. Due to the fact that the ATCN ceramic tool had higher cutting temperatures (see Figure 4), the chip will have serious friction on the flank face and cause notch wear. The boundary wear was caused by the large temperature gradient at the boundary and the severe friction of hard points at the boundary. As shown in Figure 7d, the flank wear of the ATCN-Z-C ceramic tool was mainly adhesive wear and slight boundary wear. The existence of solid lubricant film can also be observed on the flank of the ATCN-Z-C ceramic tool, and the wear area was flat. In addition, the fracture toughness of the ATCN-Z-C ceramic tool was higher than the ATCN ceramic tool, so the wear degree of the flank of the ATCN-Z-C ceramic tool was better than the ATCN ceramic tool.

**Figure 8:**
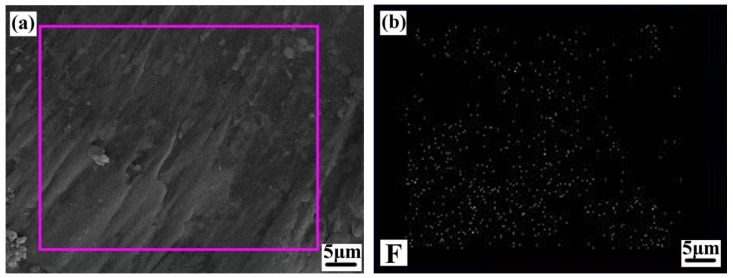
(**a**) High magnification scanning electron microscopy (SEM) micrographs of the rake face of the ATCN-Z-C tool and (**b**) the F element distribution.

Figure 8 shows a high magnification SEM micrograph of the rake face of the ATCN-Z-C ceramic tool. As shown in Figure 8a, the lubricating film can be clearly seen. In Figure 8b, the distribution of F elements can be seen. The results show that CaF_2_@Al_2_O_3_ particles were damaged during cutting, and CaF_2_ drags on the rake face to form a solid lubricating film. In the cutting process, the solid lubricating film was continuously destroyed and formed after being destroyed, so that the ATCN-Z-C ceramic tool can be continuously subjected to the wear reduction and wear resistance effects of the solid lubricating film. Due to the low shear strength of the solid lubricating film, the wear of the ATCN-Z-C ceramic tool can be well reduced during the cutting process. The existence of solid lubricating film reduced the cutting temperature and tool wear of the ATCN-Z-C ceramic tool, and improved the anti-chipping property of the ATCN-Z-C ceramic tool. In addition, the existence of solid lubricant film alleviated the stress gradient and temperature gradient at the boundary, thus reducing the boundary wear of the flank of the ATCN-Z-C ceramic tool. 

## 4. Conclusions

In this paper, CaF_2_ was prepared by the reaction of NH_4_F and Ca(NO_3_)_2_, and a layer of Al(OH)_3_ was coated on the surface of CaF_2_ by the heterogeneous nucleation method. An Al_2_O_3_/Ti(C,N) ceramic tool with CaF_2_@Al(OH)_3_ particles and ZrO_2_ whiskers was prepared. The effects of adding CaF_2_@Al(OH)_3_ particles and ZrO_2_ whiskers on the mechanical properties and cutting properties of the ceramic tool were analyzed. The following conclusions follow:

(1) Adding CaF_2_@Al(OH)_3_ particles and ZrO_2_ whiskers can increase the mechanical properties of the ceramic tool. Compared with the Al_2_O_3_/Ti(C,N) ceramic tool, the fracture toughness of the Al_2_O_3_/Ti(C,N)/ZrO_2_/CaF_2_@Al(OH)_3_ ceramic tool increased by 19.27%, but the hardness and strength decreased, and the strength decreased less.

(2) The addition of CaF_2_@Al(OH)_3_ particles and ZrO_2_ whiskers improves the cutting performance of this ceramic tool. Compared with the Al_2_O_3_/Ti(C,N) ceramic tool, this Al_2_O_3_/Ti(C,N)/ZrO_2_/CaF_2_@Al(OH)_3_ ceramic tool has lower cutting temperature and surface roughness. When the cutting speed was 300 m/min, the Al_2_O_3_/Ti(C,N)/ZrO_2_/CaF_2_@Al(OH)_3_ ceramic tool shows less flank wear than the Al_2_O_3_/Ti(C,N) ceramic tool. 

(3) The main wear mechanisms of the rake face of the Al_2_O_3_/Ti(C,N)/ZrO_2_/CaF_2_@Al(OH)_3_ ceramic tool were adhesive wear and micro-chipping, and the flank wear was adhesive wear. The presence of solid lubricating film and high toughness were important factors for the excellent wear resistance of our Al_2_O_3_/Ti(C,N)/ZrO_2_/CaF_2_@Al(OH)_3_ ceramic tool. 

## Figures and Tables

**Table 1:** Composition of workpiece material 40Cr (wt %).

Workpiece	C	Si	Mn	Cr	Ni	S	P	Fe
40Cr	0.37–0.45	0.17–0.37	0.5–0.8	0.8–1.1	≤0.03	≤0.035	≤0.035	Bal.

**Table 2:** The mechanical property of ceramic tool materials.

Tools	Compositions (vol %)	Flexural Strength (MPa)	Fracture Toughness (MPa·m^1/2^)	Hardness (GPa)
ATCN	Al_2_O_3_/Ti(C,N)	555 ± 16.65	5.78 ± 0.17	20.47 ± 0.61
ATCN-Z-C	Al_2_O_3_/Ti(C,N)/6vol%ZrO_2_/10vol%CaF_2_@Al(OH)_3_	540 ± 16.2	7.16 ± 0.21	16.72 ± 0.50
